# An inhibitor of BRD4, GNE987, inhibits the growth of glioblastoma cells by targeting C-Myc and S100A16

**DOI:** 10.1007/s00280-022-04483-7

**Published:** 2022-10-12

**Authors:** Liya Ma, Gen Li, Tianquan Yang, Li Zhang, Xinxin Wang, Xiaowen Xu, Hong Ni

**Affiliations:** 1 Institute of Pediatric Research, Children’s Hospital of Soochow University, Suzhou, 215003 People’s Republic of China; 2 Medical College of Soochow University, Suzhou, 215123 Jiangsu People’s Republic of China; 3 Department of Neurosurgery, Children’s Hospital of Soochow University, Suzhou, 215003 People’s Republic of China

**Keywords:** GNE987, BRD4, Glioblastoma, C-Myc, H3K27Ac, S100A16

## Abstract

**Purpose:**

Among children, glioblastomas (GBMs) are a relatively common type of brain tumor. BRD4 expression was elevated in GBM and negatively correlated with the prognosis of glioma. We investigated the anti-GBM effects of a novel BRD4 inhibitor GNE987.

**Methods:**

We evaluated the anti-tumor effect of GNE987 in vitro and in vivo by Western blot, CCK8, flow cytometry detection, clone formation, the size of xenografts, and Ki67 immunohistochemical staining, and combined ChIP-seq with RNA-seq techniques to find its anti-tumor mechanism.

**Results:**

In vitro experiments showed that GNE987 significantly degraded BRD4, inhibited the proliferation of GBM cells, blocked the cell cycle, and induced apoptosis. Similarly, in vivo experiments, GNE987 also inhibited GBM growth as seen from the size of xenografts and Ki67 immunohistochemical staining. Based on Western blotting, GNE987 can significantly reduce the protein level of C-Myc; meanwhile, we combined ChIP-seq with RNA-seq techniques to confirm that GNE987 downregulated the transcription of S100A16 by disturbing H3K27Ac. Furthermore, we validated that S100A16 is indispensable in GBM growth.

**Conclusion:**

GNE987 may be effective against GBM that targets C-Myc expression and influences S100A16 transcription through downregulation of BRD4.

**Supplementary Information:**

The online version contains supplementary material available at 10.1007/s00280-022-04483-7.

## Introduction

Gliomas, primary intracranial tumors with the highest incidence, account for > 30% of all central nervous system tumors. WHO classifies glioma into four grades: astrocytomas, oligodendrogliomas, or oligoastrocytomas according to the histopathological lineage [1]. The incidence of glioblastoma (WHO grade IV) is 3–5 per 100,000 [2]. Chemotherapy resistance, tumor heterogeneity, and infiltration patterns of GBM make multimodal therapy for GBM still ineffective. Chemotherapy resistance exacerbates glioma patients’ recurrence and low survival rate [3]. Therefore, it is necessary to discover new chemotherapeutic drugs to improve the treatment of human GBM.

Several studies found that BRD4 is highly expressed in GBM [4, 5]. To date, various BRD4 targeted inhibitors have been developed. However, most BRD4 inhibitors have problems, such as inaccurate targeting, off-target effects, short half-life, and drug resistance. Therefore, some scientists have developed a new technique called proteolysis-targeting chimeras (PROTACs) that utilizes the cell's ubiquitin–proteasome system (UPS) to solve these problems. PROTAC is a heterofunctional bi-specific molecule. Three components are included in PROTAC: a target-specific ligand, an E3 ubiquitin ligase binder, and a linker that links the two together [6]. GNE987 is a PROTAC linked by von Hippel-Lindau (VHL) and BRD4 ligands. GNE987 reduces cell growth and increases apoptosis in neuroblastoma [7] and AML [8]. However, its anti-tumor activity and underlying mechanism in GBM are unavailable to date.

C-Myc is a well-studied oncogene whose upregulation has been demonstrated in various cancers, including gastric [9], breast [10], and GBM [11]. A recent study demonstrated that GNE987 rapidly and persistently degrades BRD4 and inhibits downstream targets such as C-Myc in neuroblastoma [7]. BRD4 belongs to the BET family, which recognizes acetylated lysine residues on histone and non-histone chromatin factors to manage gene expression [12, 13]. Acetylation of histone H3 at K27 (H3K27Ac) is used to annotate actively transcribed chromatin regions. Therefore, this study used H3K27Ac ChIP-seq combined with RNA-seq technology to explore the molecular mechanism of action of GNE987 against GBM.

## Materials and methods

### Cell cultures

The human GBM cell lines U87(RRID: CVCL_0022), LN229 (RRID: CVCL_0393), U251 (RRID: CVCL_0021), and A172 (RRID: CVCL_0131) and human embryonic kidney cell line (293FT, RRID: CVCL_6911) were purchased from ATCC. The cells were cultured in DMEM supplemented with 10% FBS, 1% penicillin, and streptomycin. All cells within passages 8 to 15 were used and passed the detection of mycoplasma contamination by Myco-Lumi^TM^Mycoplasma Kit (Beyotime, China).

### Lentivirus preparation and infection

The shRNA and negative control of VHL were constructed in the pLKO.1 vector, and the overexpression and negative control of VHL were constructed in the PLVX-Flag vector (IGEbio,China). The shRNA and negative control S100A16 were constructed in the Tet-pLKO vector (IGEbio,China), and DOX (Sigma, USA) at 1 μg/mL induced shRNA expression. The sequences of the knockdown genes were as follows:

- The VHL shRNA targeting sequence: 5′-CCG GGC TCA ACT TCG ACG GCG AGC CCT CGA GGG CTC GCC GTC GAA GTT GAG CTT TTT TGA ATT-3′;

The S100A16 shRNA targeting sequences are listed below:

- S100A16 shRNA-1: 5′-CAG TCA TTG TCC TGG TGG AAA TTT CCA CCA GGA CAA TGA CTG-3′;
- S100A16 shRNA-2: 5′-CGA TGA GTA CTG GAC CTT GAT ATC AAG TCC AGT ACT CAT CG-3′;
- S100A16 shRNA-3: 5′-CAG CCT GGT CAA GAA CAA GAT ATC TTG TTC TTG ACC AG -3′;

1 μg/mL Puromycin (Beyotime, China) was used to screen stable strains of cells.

### Cell viability and proliferation assay

GBM cells (2 × 10^3^/well) were cultured overnight in 96-well plates; GBM cells were treated with GNE987, JQ1, ARV825, dBET1 (MedChemExpress, USA) or DMSO (Sigma, USA) for 3 days,5 days or 7 days, and the absorbance of a 96-well plate was measured using a microplate reader with CCK8 (Dojindo, Japan). Cell viability and proliferation rates were calculated using GraphPad Prism8.4.0.

### Clone formation assay

GBM cells (1 × 10^3^/well) were seeded into 6-well plates, and DMSO or GNE987 was incubated for 2 weeks. GBM cells were washed with PBS and fixed with methanol and stained with Giemsa (Solarbio, China). Pictures were taken and clones were counted.

### Cell cycle analysis

GBM cells (20 × 10^4^/well) were seeded into 6-well plates, and DMSO or GNE987 was incubated for 3 days. Cold 70% ethanol was applied overnight to GBM cells, followed by one wash with PBS the next day. Light-free incubation using the cell cycle analysis kit (Cat#C1052, Beyotime, China) for 30 min. Flow cytometry (Beckman Gallios, USA) was used to test the cells.

### Cell apoptosis assay

GBM cells (20 × 10^4^/well) were seeded into 6-well plates, and DMSO or GNE987 was added and incubated for 3 days. And GBM cells were stained using the FITC-Annexin V Apoptosis Kit (Cat#556547, BD, USA). Flow cytometry (Beckman Gallios, USA) was used to test the cells and analyzed to determine the proportion of apoptotic cells.

### EdU staining analysis

GBM cells (5 × 10^4^/well) were seeded into 24-well plates and treated with GNE987 or DMSO for 3 days, then stained by the EdU staining kit (BeyoClick™ EdU-488 Cell Proliferation Assay kit, Beyotime, China) according to a previous protocol [14]. Cells were treated with EdU working solution for 2 h, 4% paraformaldehyde for about 10 min, 3% BSA for 1 h, and then A 30 min click reaction at room temperature away from light, and DAPI for 5 min.

### In vivo xenografts

For the GBM subcutaneous transplanted tumor model, 5 × 10^6^ U87 cells were inoculated in the left- back of nude mice (Shanghai ling chang biotech, 4 weeks of age, female, *n* = 6/group), and tumor-bearing mice were randomly divided into two groups, the drug treatment group, and the vehicle group. The day the tumor was received was defined as day 0, from the 3rd day after the tumor, and the drug treatment group was given an intraperitoneal injection of GNE987 (0.25 mg/kg) every 2 days according to a previous protocol [7]; the vehicle group was administered the same dose of 5% Kolliphor®HS15 as GNE987. The mice were weighed and measured every two to three days to determine their body weight and tumor volume. The survival endpoint was defined as when the tumor in the vehicle group exceeded 1 cm^3^. The Animal Ethics Committee of Soochow University approved this research (CAM-SU-AP#: JP-2018-1).

### Immunohistochemistry (IHC)

IHC was performed using the IHC Kit (Cat#KIT-9720, MXB Biotechnology, China). Sections were subjected to dewaxing, hydration, antigen retrieval, Ki67 antibody (Cat. ab15580, Abcam, UK), DAB chromogenic reaction (Cat#DAB-2031, MXB Biotechnology, China), hematoxylin staining (Beyotime, China), and dehydration. Brown-yellow indicated positive expression, and violet-blue indicated the nucleus.

### RT-qPCR

Cells were harvested using TRIzol reagent (Invitrogen, USA), total RNA was extracted using Chloroform reagent, isopropanol reagent, and cDNA was synthesized according to a previous protocol [7]. RT-qPCR was performed using LightCycler 480 Real Time System (Roche, Germany). The relative mRNA expression was calculated using the 2^–ΔΔCT^ method. Glyceraldehyde-3-Phosphate dehydrogenase (GAPDH) was used for interior management. The primer sequences used were as follows:

- *GAPDH*: 5′-ATC ATC CCT GCC TCT ACT GG-3′ (forward) and 5′-CCC TCC GAC GCC TGC TTC AC-3′ (reverse).
- *cyclinB*: 5′ TCG CCT GAG CCT ATT TTG GT-3′ (forward) and 5′-GCA TCT TACT TGG GCA CAC AA-3′ (reverse).
- *cdc2*: 5′ AGT CTG GTC TTT CTT TGG CTG TCA G-3′ (forward) and 5′-AAA CAC CTA CAA CCA CCA CTC TGC-3′ (reverse).
- *WNT5A*: 5′-TAC GAG AGT GCT CGC ATC CTC A-3′ (forward) and 5′-TGT CTT CAG GCT ACA TGA GCC G-3′ (reverse).
- *ZMYND8*: 5′-AAG CGC CAG ATT CGT AGC AGG T-3′ (forward) and 5′-TCC TCC GAA TCG CTG TGC TCT A-3′ (reverse).
- *BCL2L1*: 5′-GCC ACT TAC CTG AAT GAC CAC C-3′ (forward) and 5′-AAC CAG CGG TTG AAG CGT TCC T-3′ (reverse).
- *CAV1*: 5′-CCA AGG AGA TCG ACC TGG TCA A-3′ (forward) and 5′-GCC GTC AAA ACT GTG TGT CCC T-3′ (reverse).
- *TBX2*: 5′-AGC AGT GGA TGG CTA AGC CTG T-3′ (forward) and 5′-GGA TGT CGT TGG CTC GCA CTA T-3′ (reverse).
- *STEAP3*: 5′-TGC AAA CTC GCT CAA CTG GAG G-3′ (forward) and 5′-AGG CAG GTA GAA CTT GTA GCG G-3′ (reverse).
- *POU2F2*: 5′-TCC TGG AGA AGT GGC TCA ACG A-3′ (forward) and 5′-ATG CTG GTC CTC TTC TTG CGT C-3′ (reverse).
- *EPHA2*: 5′-ACT GCC AGT GTC AGC ATC AAC C-3′ (forward) and 5′-GTG ACC TCG TAC TTC CAC ACT C-3′ (reverse).
- *KCNJ15*: 5′-TGT GCT TGG TGA TTC AGG TAG CC-3′ (forward) and 5′-GAC AGT GGC TTG GTT GAG GAG A-3′ (reverse).
- *EPSTI1*: 5′-ACT GAA ACG GCA GCA GCA AGA G-3′ (forward) and 5′-TCC AAC AGC CTC CAG ATT GCT C-3′ (reverse).
- *PALMD*: 5′-GAG GAA GAC AAA CTA AAG CAC CAG-3′ (forward) and 5′-CTC TTC CTG TTC TTT TCC GCT GC-3′ (reverse).
- *S100A16*: 5′-GCT CCA GAA AGA GCT GAA CCA C-3′ (forward) and 5′-ATG CCG CCT ATC AAG GTC CAG T-3′ (reverse).

### Western blot

GBM cells were treated with GNE987 or DMSO for 48 h. Then, proteins were extracted with RIPA supplemented with protease and phosphatase inhibitors, Western blot was performed using appropriate primary and secondary antibodies. The specific antibodies included the following: BRD4 (Cat#13440 s, CST, USA), BRD2 (Cat#5848 s, CST, USA), β-TUBULIN (Cat#2146, CST, USA), FLAG (Cat#14793S, CST, USA), VHL (Cat#68547S, CST, USA) and C-Myc (Cat#9402; CST, USA), BRD3 (Cat#11859-1-AP, Proteintech, USA), and GAPDH (Cat# MA3374, Millipore, USA).

### RNA sequencing analysis

For RNA-seq (Novogene Ltd., China), U87 cells were treated with GNE987 and DMSO for 48 h, and then harvested using TRIzol reagent (Invitrogen, USA). Differentially expressed genes were identified by the Bioconductor DESeq2.

### Chromatin immunoprecipitation sequencing (ChIP-seq) data processing

ChIP was performed according to a previous protocol [7]. First, 3 × 10^7^ U87 cells were cross-linked with 1% paraformaldehyde at room temperature for 10 min, neutralized with 0.125 M at room temperature for 5 min, centrifuged to collect the cells, and lysed with cell lysis buffer on ice for 5 min; cells were lysed by repeatedly aspirating the solution with an insulin needle. The solution was centrifuged, pellets were resuspended in shearing buffer, and genomic DNA was cleaved into fragments of approximately 500 bp using a sonicator (M220, Covaris, USA). After centrifugation, the supernatant was collected and the H3K27Ac antibody (Cat. ab4729, Abcam, UK) was added to it overnight at 4 °C; the next day, Dynabeads Protein G beads (Thermo Fisher Scientific, USA) were added to the supernatant at 4 °C for 4 h for immunoprecipitation. The magnetic beads were washed with TE buffer (Sigma, USA); EB buffer was added, and shaken for 15 min to separate the magnetic beads, antibodies, proteins, and DNA; the supernatant was taken, and 5 M NaCl was added to it, and the mixture was heated at 65 °C overnight. The antibodies, proteins, and DNA were separated using a PCR purification kit (QIAGEN, Germany). ChIP-seq was provided by BGI Ltd (China).

### Statistical analysis of data

Data and graphs were processed using GraphPad Prism (version 8.4.0, USA). Comparison between the two groups was performed by Student’s *t* test. ANOVA was used for comparison between multiple groups. In the graphs, mean ± standard deviation (SD) is represented; *P* value < 0.05 denoted statistical significance (*P < 0.05, **P < 0.01, ***P < 0.001).

## Results

### BRD4 is overexpressed in patients with glioma

**Fig. 1:**
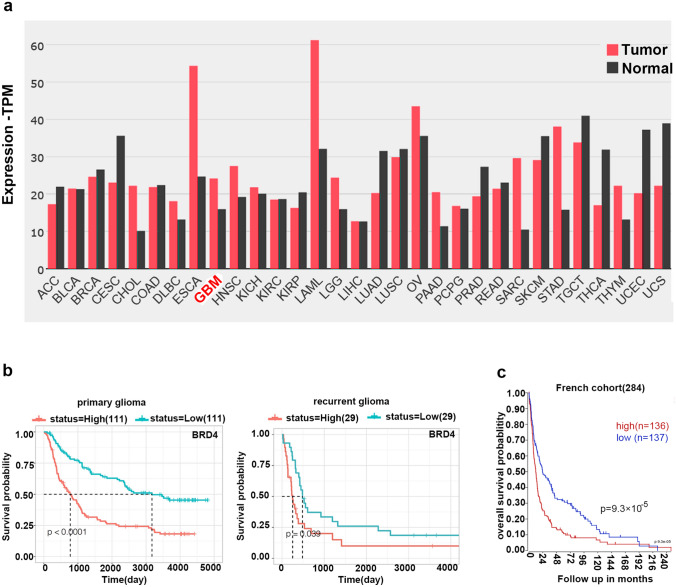
BRD4 is overexpressed in patients with glioma. **a** Expression of BRD4 mRNA in tumors and normal tissues (source: GEPIA2: http://gepia2.cancer-pku.cn). **b** Survival curves of high and low BRD4 expression in 222 and 58 patients with primary and recurrent glioma, respectively (http://www.cgga.org.cn/, source: CGGA database, mRNAseq-325 Dataset). **c** Survival curve of high(red) or low(blue) BRD4 of patients with glioma (http://r2.amc.nl). The median is the cut-off point for high or low BRD4 expression

We assessed BRD4 expression in cancer and the correlation between BRD4 mRNA expression and prognosis using public databases. First, GEPIA2 data found high levels of BRD4 expression in a subset of cancers, including GBM (Fig. 1a). We analyzed the correlation between BRD4 expression and overall survival. In the CGGA database, high expression of BRD4 was found to negatively correlate with the prognosis of primary and recurrent gliomas in the mRNAseq-325 dataset (Fig. 1b). On the R2 platform, from the GEO database, a public dataset of 284 patients (GSE16011), the survival curve in Fig. 1c suggested that patients with higher BRD4 mRNA levels had shorter survival time (*P* = 9.3 × 10^–5^). These results indicated that BRD4 may be a vital therapeutic target in GBM.

### GNE987 damages the viability of GBM cells and inhibits cell proliferation

**Fig. 2:**
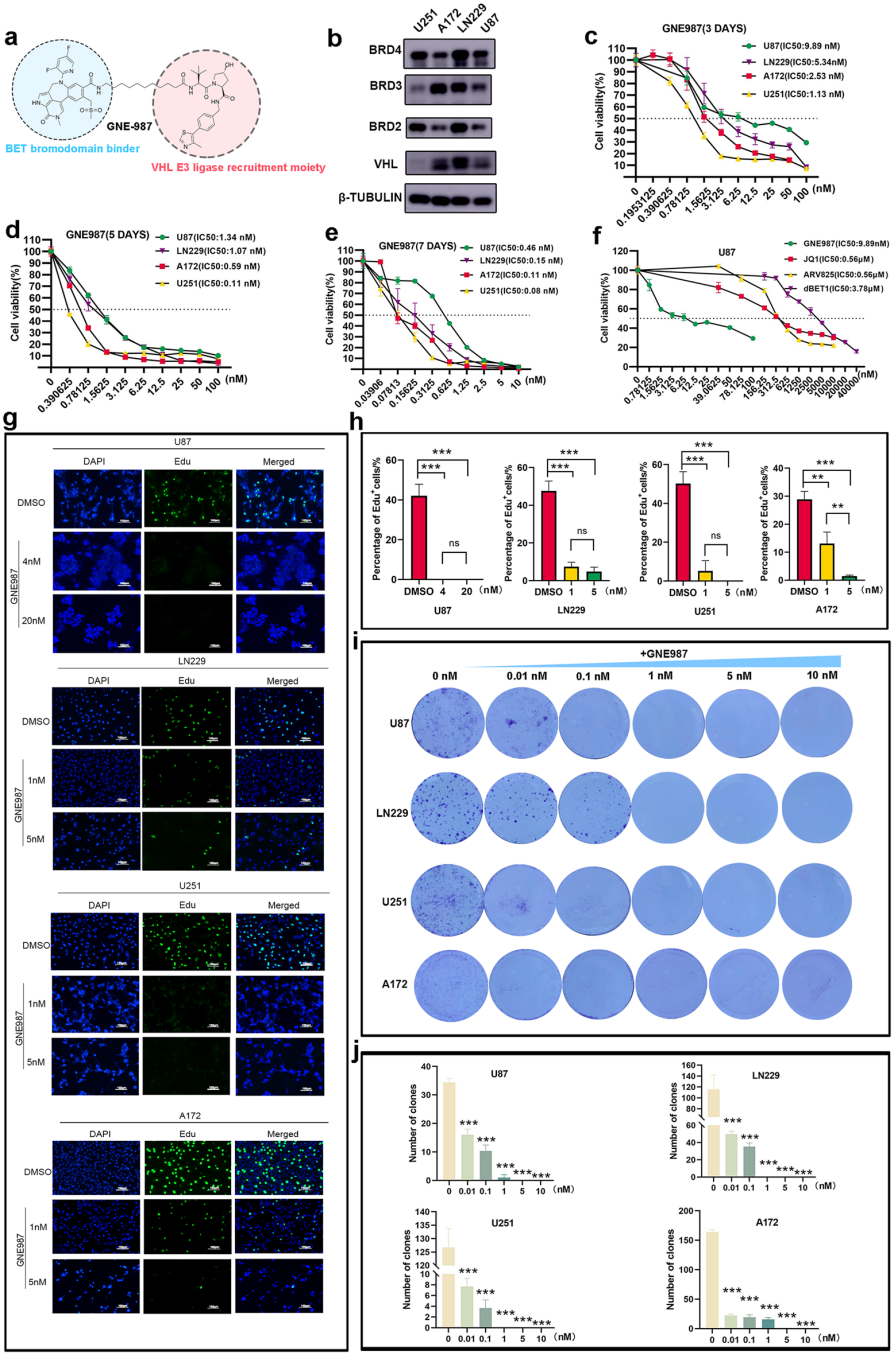
GNE987 damages the viability of GBM cells and inhibits cell proliferation. **a** Schematic diagram of bifunctional PROTAC molecules. **b** BET and VHL protein levels in GBM cells. **c** The IC50 value of GNE987 at 3 days in GBM cell lines. **d** The IC50 value of GNE987 at 5 days in GBM cell lines. **e** The IC50 value of GNE987 at 7 days in GBM cell lines. **f** Various concentrations of GNE987, JQ1, ARV825 and dBET1 affect cell viability for 3 days in U87 cells. The IC50 value of GNE987, JQ1, ARV825 and dBET1 in U87 cell line. **g** EdU staining of GBM cells treated with DMSO or GNE987 for 3 days; White bar, 100 μm. **h** The bar graph shows the percentage of positive cells of EdU. **i** Clone-forming ability of DMSO group and GNE987 groups. **j** Bar graph of the colony-forming ability of GBM cells treated with DMSO or GNE987. (Data were presented with mean ± SD of three independent experiments, *P < 0.05, **P < 0.01, ***P < 0.001)

GNE987 is a PROTAC linked by a VHL and BRD4 ligand. Its chemical structure is shown (Fig. 2a). BRD4, BRD2, BRD3, and VHL were all expressed in U87, LN229, U251, and A172 (Fig. 2b), implying that BET and VHL proteins are widely expressed in GBM cells; The effect of treatment, with different GNE987 doses for 3 days, 5 days and 7 days, on the viability of GBM cell lines was assessed using CCK8. The result is that the IC50 of GNE987 at 3 days is 9.89 nM, 5.34 nM, 1.13 nM, 2.53 nM in U87, LN229, U251 and A172, respectively; The IC50 of GNE987 at 5 days is 1.34 nM, 1.07 nM, 0.11 nM,0.59 nM in U87, LN229, U251 and A172; The IC50 of GNE987 at 7 days is 0.46 nM, 0.15 nM, 0.08 nM,0.11 nM in U87, LN229, U251 and A172, which demonstrated that GNE987 extensively inhibited the viability of GBM cells in a dose-dependent and time-dependent manner (Fig. 2c–e). Meanwhile, to demonstrate the dose advantage of GNE987 over other BRD4 inhibitors, we evaluated the IC50 of JQ1, ARV825, and dBET1 at 3 days in U87 cells and found that the IC50 of GNE987(9.89 nM) was much smaller than these three BRD4 inhibitors (JQ1 0.56 μM, ARV825 0.56 μM, dBET1 3.78 μM) (Fig. 2f). EdU staining showed that GNE987 significantly inhibited cell proliferation (Fig. 2g, h). GNE987 inhibited colony formation in a dose-dependent manner in GBM cells (Fig. 2i, j). These findings demonstrated that GNE987 exerts an antiproliferative effect on GBM cells.

### GNE987 induces cell apoptosis, arrests cell cycle and decreases BRD4 protein levels

**Fig. 3:**
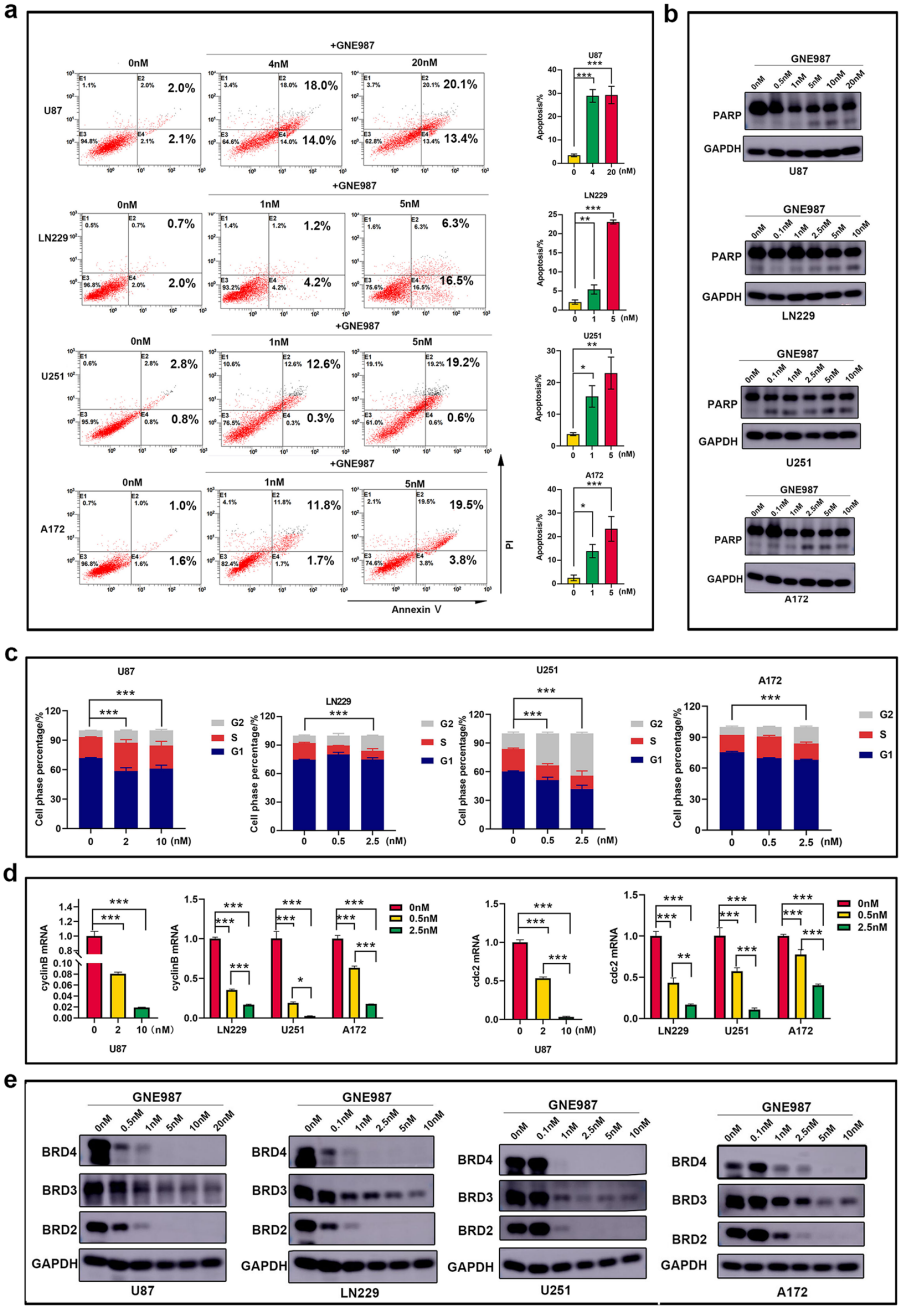
GNE987 induces cell apoptosis, arrests cell cycle and decreases BRD4 protein levels. **a** The apoptosis rate of GNE987 was increased through Annexin-V/PI staining. **b** GNE987 induced the emergence of cleaved-PARP in GBM cells. **c** The cell cycle assay showed that the G2 phase increased after GNE987 treatment. **d** cyclinB and cdc2 mRNA levels after DMSO or GNE987 treatment. **e** GNE987 strongly decreased BET protein levels in GBM cells (Data were presented with mean ± SD of three independent experiments, *P < 0.05, **P < 0.01, ***P < 0.001)

Efficient elimination of cancer cells through programmed cell death or apoptosis has long been the goal of clinical cancer therapy [15]. GBM cell apoptosis was also detected after GNE987 treatment by flow cytometer (Fig. 3a), and Western blotting analysis showed that GNE987 treatment increased the cleaved-PARP (Fig. 3b). GNE987 arrested the cell cycle. Compared to the control group, GNE987 increased the proportion of the G2 phase (Fig. 3c). The mRNA levels of *cyclinB* and *cdc2*, which are cell cycle-related genes, decreased in a dose-dependent manner through RT-qPCR verification (Fig. 3d). These findings demonstrated that GNE987 has a strong ability to induce cell apoptosis and cell cycle arrest. Western blotting analysis showed that GNE987 decreased BRD4 protein levels in GBM cells. Furthermore, GNE987 can reduce both BRD2 and BRD3, but is more sensitive to BRD4 than BRD2 and BRD3 (Fig. 3e).

### VHL expression correlates with the anti-tumor effect of GNE987

**Fig. 4:**
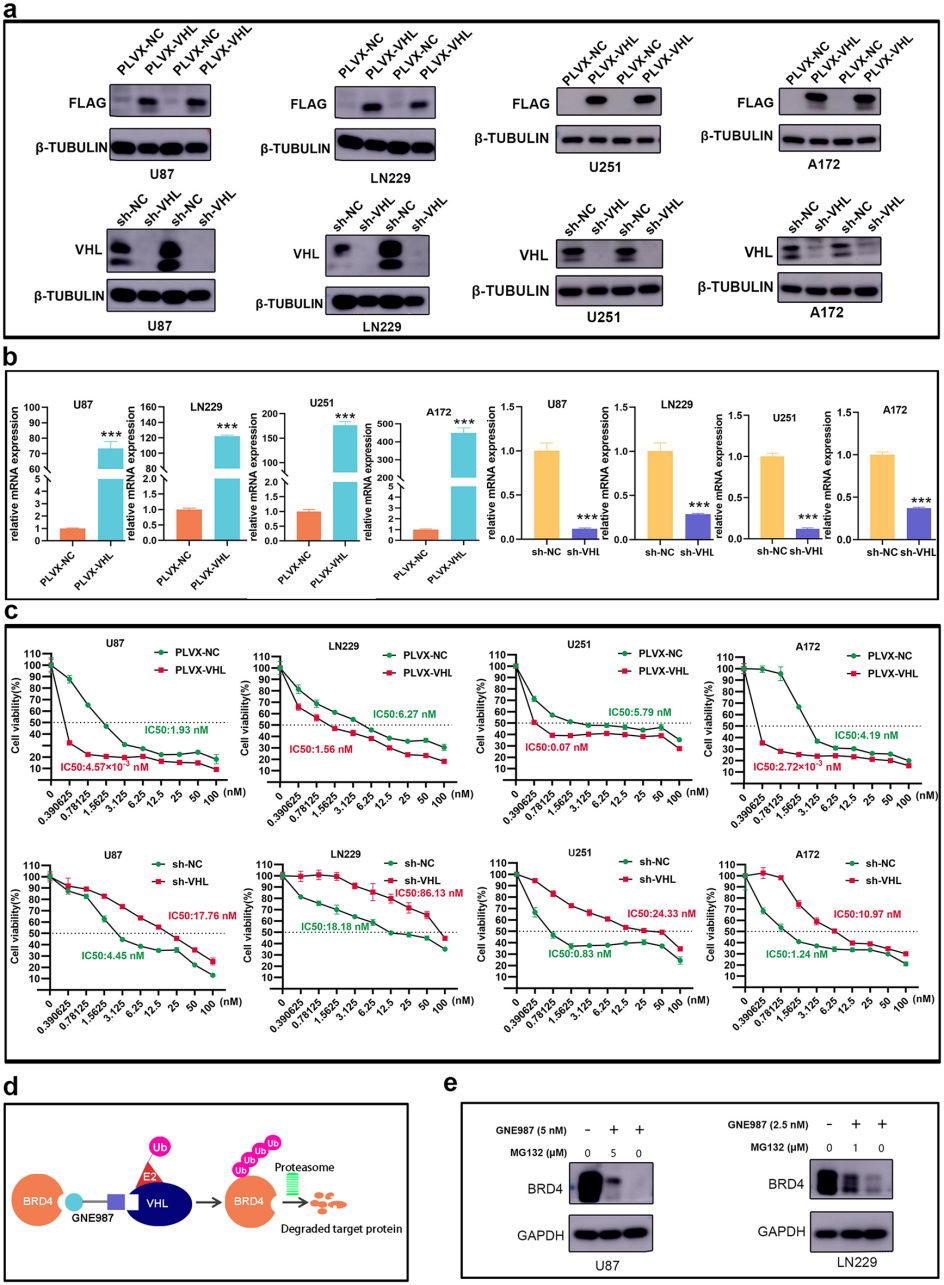
GNE987's anti-tumor activity is closely related to VHL expression. **a** Identification of VHL overexpression or knockdown by Western blotting in GBM cells. **b** Identification of VHL overexpression or knockdown by RT-qPCR in GBM cells. **c** Effects of VHL overexpression or knockdown on cell viability after treatment of GBM cells with GNE987. **d** Schematic diagram of the targeted degradation of BRD4 by GNE987; GNE987 binds both BRD4 and a VHL E3-ubiquitin ligase complex. Formation of the trimeric complex results in the transfer of ubiquitin to BRD4. **e** Western blotting of BRD in U87 and LN229 cells treated with GNE987, MG132 and their combination. (Data were presented with mean ± SD of three independent experiments, *P < 0.05, **P < 0.01, ***P < 0.001)

VHL is the E3 ubiquitin ligase [16]. To further evaluate the relationship between VHL expression levels and sensitivity of GBM cell lines to GNE987 treatment, we constructed the stable strains of VHL overexpression or knockdown in GBM cell lines, and identified them with Western blot and RT-qPCR (Fig. 4a, b). CCK8 assay revealed that VHL overexpression significantly increased the sensitivity of GBM cells to GNE987; however, its knockdown partially reduced growth inhibition by GNE987 in GBM cells (Fig. 4c). The schematic diagram shows that GNE987 drives polyubiquitination of BRD4 by the E3 ubiquitin ligase complex by linking VHL and BRD4; subsequently, BRD4 polyubiquitination is recognized by the proteasome and digested into amino acids and small peptides (Fig. 4d). An inhibitor of the proteasome, MG132 (Beyotime), was used to determine the role of the proteasome in GNE987-induced BRD4 degradation. Western blot revealed that degradation of BRD4 protein by GNE987 was partially rescued by MG132, which demonstrates that GNE987 partially depends on ubiquitination to degrade BRD4 in U87 and LN229 cells (Fig. 4e). These findings demonstrated that VHL plays an important role in the growth-inhibitory activity of GNE987.

### GNE987 inhibits tumor growth of GBM in vivo experiments

**Fig. 5:**
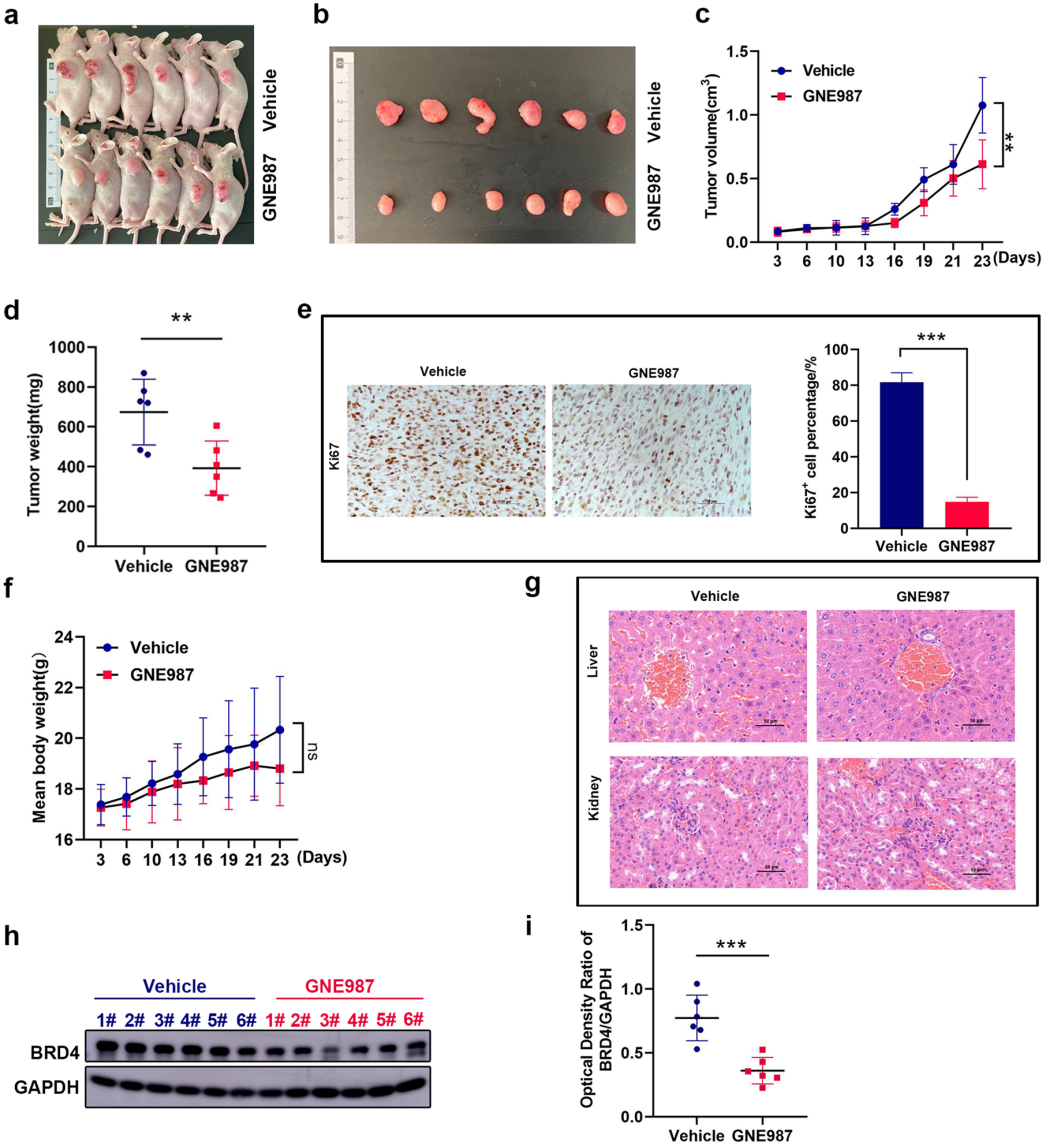
GNE987 inhibits tumor growth of GBM in vivo experiments. **a** Photo of tumor-bearing mice. **b** Tumor photos of the vehicle and GNE987 groups. **c** Growth curve of tumor volume; the formula for tumor volume is: (length × width^2^)/2. **d** Tumor weights in the vehicle group (blue) and GNE987 group (red). **e** Ki67 expression in the vehicle group and GNE987 group; brown-yellow denotes positive expression and purple-blue denotes the cell nucleus (Scale bar is 50 µm). **f** Body weight growth curve of tumor-bearing mice in the vehicle and GNE987 groups. **g** H&E staining of the liver and kidney of tumor-bearing mice in the vehicle and GNE987 groups (Scale bar is 50 µm). **h** GNE987 was injected every 2 days from the 3rd day, and the samples were harvested 48 h after the last dose of GNE987 on the 21st day for Western blot detection. Compared to the vehicle group (blue), BRD4 expression in tumors of the GNE987 group (red) was significantly decreased. **i** The levels of BRD4 protein in the vehicle group (blue) and GNE987 group (red). (n.s., not significant, **P < 0.01, ***P < 0.001, *n* = 6)

Using U87 cells, xenograft models of GBM were established to study the anti-tumor effect of GNE987 in vivo (Fig. 5a, b). Compared to the vehicle group, the tumor volume, tumor mass, and proliferation marker (Ki67) were significantly decreased in the GNE987-treated group (Fig. 5c–e). In Fig. 5f, g, GNE987 caused a certain downward trend in body weight, but it had no obvious side effects on the liver and kidney. Furthermore, we determined that GNE987 could also decrease the protein levels of BRD4 in vivo (Fig. 5h, i).

### Mechanism of anti-tumor effect of GNE987

**Fig. 6:**
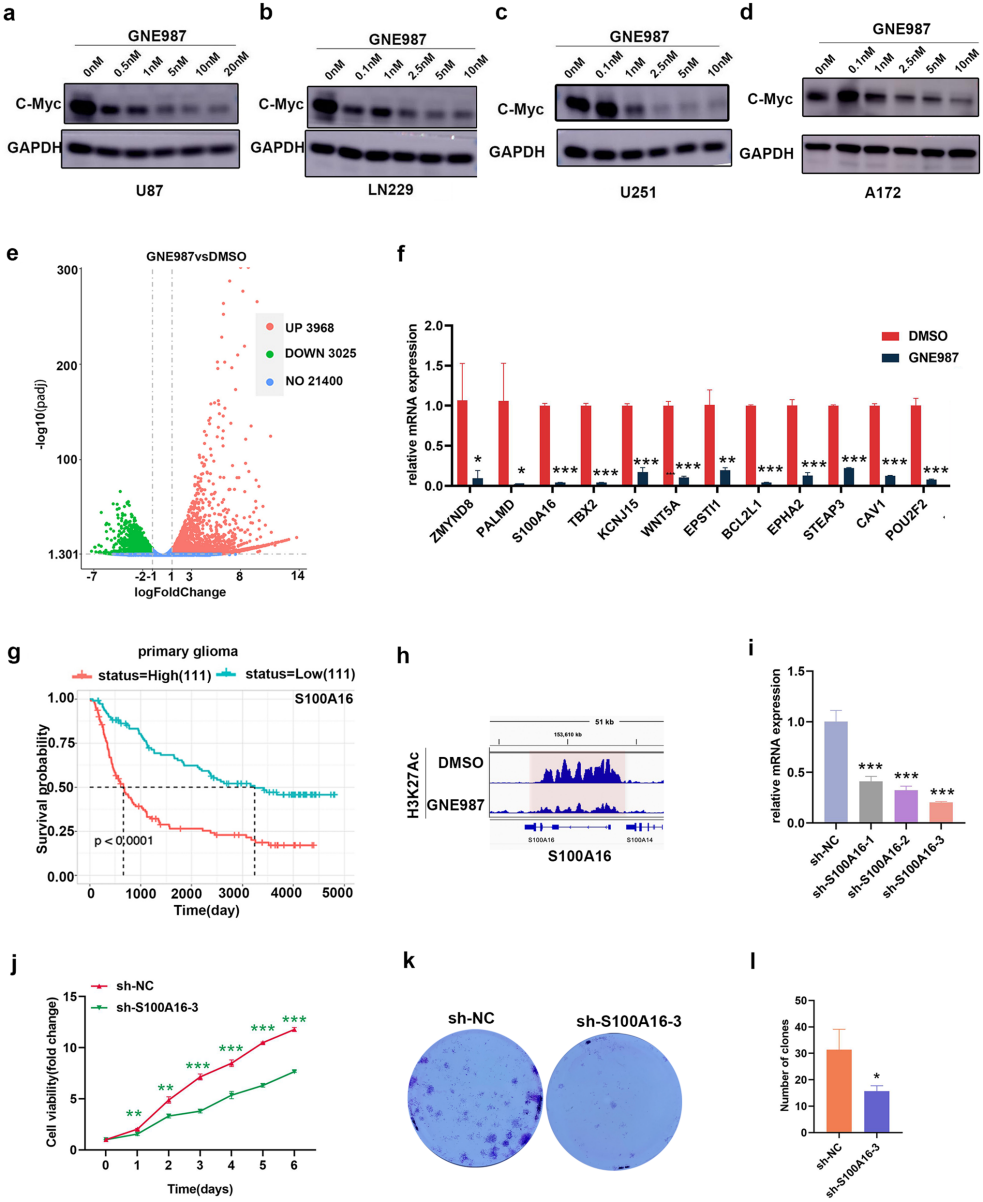
Mechanism of anti-tumor effect of GNE987. **a**–**d** GNE987 reduces C-Myc expression in U87 cells, LN229 cells, U251 cells and A172 cells. **e** Volcano plot shows the differential genes from the GNE987 group or DMSO group (|log2FoldChange|> 1, adjusted *p* < 0.05). **f** mRNA levels of tumor-related genes, *WNT5A*,* ZMYND8*,* BCL2L1*,* CAV1*,* TBX2*,* STEAP3*, *POU2F2*,* EPHA2*,* KCNJ15*,* EPSTI1*,* PALMD*, and *S100A16,* were significantly downregulated after GNE987 treatment. **g** S100A16 expression level closely correlates with survival probability in patients with primary glioma (Source: CGGA database, mRNAseq-325 dataset). **h** IGV view software displays gene tracks of H3K27Ac ChIP-seq occupancy at S100A16 gene loci from the GNE987 group or DMSO group; the *x*-axis shows the genomic location and the *y*-axis reflects H3K27Ac enrichment. **i** RT-qPCR analysis of the knockdown efficiency of shRNA of the GNE987-sensitive gene, S100A16. **j** Effects of S100A16 knockdown on proliferation. **k**, **l** Effects of S100A16 knockdown on clone formation (Data were presented with mean ± SD of three independent experiments, *P < 0.05, **P < 0.01, ***P < 0.001)

C-Myc is an oncogene signaling pathway that can regulate biological processes, such as apoptosis, proliferation, survival, and differentiation. GNE987 decreased C-Myc protein levels in GBM cells (Fig. 6a–d).

To identify other underlying mechanisms of action of GNE987, genes were screened and analyzed using RNA-seq. As shown, the mRNA levels of 3,968 genes were elevated and those of 3,025 genes were decreased in GNE987-treated U87 cells, compared to controls with |log2fold change|> 1 and adjusted *p* < 0.05 (Fig. 6e). To confirm the reliability of the RNA-seq results, we selected several genes for verification. As expected, RT-qPCR results confirmed that the mRNA levels of tumor-related genes, *WNT5A, ZMYND8, BCL2L1, CAV1, TBX2, STEAP3, POU2F2, EPHA2, KCNJ15, EPSTI1, PALMD,* and *S100A16* were significantly downregulated after GNE987 treatment in U87 cells. (Fig. 6f). The expression abundance of S100A16 significantly correlated with the survival probability of glioma patients (Fig. 6g); moreover, H3K27Ac can be used to annotate transcriptionally active chromatin regions [17]; thus, we obtained H3K27Ac ChIP-seq data to map the gene locus of S100A16, and visualized using IGV View Software to characterize the effect of GNE987 on S100A16 (Fig. 6h). The results showed that GNE987 significantly reduced the enrichment of H3K27Ac at the S100A16 locus, suggesting that GNE987 downregulated S100A16 by disturbing H3K27Ac.

To verify the anti-GBM function of S100A16, we used shRNA-mediated knockdown of S100A16 in U87 cells (Fig. 6i) and observed the effect of the candidate oncogene, S100A16, on cell viability using CCK8. The results showed that S100A16 knockdown significantly disrupted U87 cell viability (Fig. 6j), and S100A16 knockdown significantly affected the colony-forming ability of U87 cells (Fig. 6k, l). These results indicated that S100A16 was able to maintain the growth and survival of GBM cells, which fully met our expectations.

## Discussion

GBM is the most lethal primary tumor of the central nervous system. Temozolomide (TMZ) acts as first-line chemotherapy drug, but its acquired resistance has become a major barrier to effectiveness [18].

Epigenetic proteins recently emerged as new anti-tumor targets [19]. BET proteins govern gene expression as epigenetic readers [20–23]. BRD4 is the most well-studied and has been implicated in various human cancers. BRD4 expression level and overall survival in glioma patients were negatively correlated in public databases. JQ1 was the first BRD4 bivalent triazole azepine inhibitor developed in 2010; however, its short half-life and drug resistance hinder further clinical application [24–26]. More than 20 BRD4 inhibitors are currently undergoing clinical trials for the treatment of hematological malignancies, solid tumors, and other diseases [25]. Over the long term, researchers have found that many of the inhibitors present severe side effects and drug resistance due to mutations in the target protein [27–29]. So PROTAC was designed to try to solve these problems, which is a novel and efficient inhibitor construction system [30].

PROTAC comprises three special elements: E3 ubiquitin ligase ligands, proteins of interest (POI) ligands, and linkers. Compared to traditional small-molecule inhibitors, the advantages of PROTAC are as follows [31–33]: (1) a low concentration is required to exert the same pharmacological effect; (2) targeting undruggable proteins; (3) overcoming drug resistance in POI caused by mutations or target upregulation; (4) precise targeting. GNE987 efficiently degraded BRD4 in vitro compared to BRD2 and BRD3. GNE987 shows promising therapeutic efficacy in hematological tumors and neuroblastoma; however, its efficacy in GBM is uncertain. In this study, GNE987 effectively attenuated the growth of GBM cells by inhibiting proliferation and accelerating apoptosis. Our findings further suggest that VHL expression plays a major role in the repression of GNE987 in these four GBM cell lines. The degradation of BRD4 by GNE987 was partially rescued by MG132 treatment. Taken together, these data suggest that GNE987 leads to BRD4 degradation via VHL-mediated proteasomal degradation, which inhibits cell growth. Some studies found that subcutaneous xenografts can effectively predict patients' response to chemotherapy drugs [34–36]. Therefore, we performed a GNE987 anti-GBM experiment using subcutaneous xenografts of nude mice. It can be observed that GNE987 decreased BRD4 and inhibited tumor growth, and the important organs of nude mice (liver and kidney) had no obvious pathological changes, which suggested that GNE987 can also target and decrease BRD4 to inhibit the growth of GBM tumors without obvious toxicity and side effects, which provides a certain theoretical basis for clinical transformation.

C-Myc is a well-studied proto-oncogene; it acts as a master regulator of cell proliferation, and its upregulation has been demonstrated in GBM [11]. In this study, we also found that GNE987 interfered with C-Myc protein level in GBM cells, indicating that in GBM, GNE987 can exert an anti-tumor effect by inhibiting the Myc pathway, similar results also appear in neuroblastoma [7].

To explore other potential mechanisms of GNE987 against GBM, we detected that GNE987 interferes with the transcription of some tumor-related genes using RNA-seq data. Among these genes, we identified S100A16 using multiple criteria, including its association with glioma, functional validation, and overall survival analysis. S100A16 is involved in various tumors, such as colorectal cancer, bladder cancer, pancreatic cancer, lung adenocarcinoma, cervical cancer, leukemia, and gastric cancer [37–43]. According to the CGGA database, S100A16 closely correlates with the survival rate of patients with primary gliomas. This is also true in low-grade gliomas [44]. H3K27Ac can be used to annotate the transcriptionally active chromatin regions [17]. In this study, we found that GNE987 directly downregulates S100A16 after analyzing ChIP-seq data. Furthermore, S100A16 knockdown significantly inhibited cell proliferation. In short, GNE987 affected the transcription of many tumor-related genes. S100A16, as one of them, was preliminarily confirmed that its downregulation affects the growth of GBM cells. In line with our findings, downregulation of S100A16 inhibits the proliferation and migration of tumor cells in pancreatic and gastric cancers [45, 46].

In conclusion, our findings suggest that GNE987 can effectively inhibit the growth of GBM cells and promote apoptosis by downregulating transcription of oncogenes, thus suggesting that GNE987 may be a good therapeutic strategy for GBM and S100A16 may be a new target against GBM.

## Supplementary Information

Below is the link to the electronic supplementary material.

Supplementary file1 (DOCX 14 KB)

## Data Availability

On reasonable request, the corresponding author will provide the data generated during and/or analyzed during this study. This manuscript's RNA-seq data have been deposited in GEO (accession number GSE202362). Enter token ijofwgqsnlcjdux into the box. This manuscript's ChIP-seq data have been deposited in GEO (accession number GSE202484). Enter token ijexmmaktlwrpkf into the box.
