# Supplementary material for: An inhibitor of BRD4, GNE987, inhibits the growth of glioblastoma cells by targeting C-Myc and S100A16
Source: Cancer Chemother Pharmacol. 2022 Oct 12;90(6):431–44. doi: 10.1007/s00280-022-04483-7 (PMC9637061; doi:10.1007/s00280-022-04483-7)
Supplement: Supplementary file 1 — Supplementary file1 (DOCX 14 KB) [file 280_2022_4483_MOESM1_ESM.docx]

**Link(s) to supporting data**

GEPIA2: <http://gepia2.cancer-pku.cn>

CGGA database, mRNAseq-325 Dataset: <http://www.cgga.org.cn>

R2 platform: <http://r2.amc.nl>
